# Cognitive Mediators of School-Related Socio-Adaptive Behaviors in ASD and Intellectual Disability Pre- and Adolescents: A Pilot-Study in French Special Education Classrooms

**DOI:** 10.3390/brainsci9120334

**Published:** 2019-11-21

**Authors:** Cécile Mazon, Charles Fage, Charles Consel, Anouck Amestoy, Isabelle Hesling, Manuel Bouvard, Kattalin Etchegoyhen, Hélène Sauzéon

**Affiliations:** 1 Equipe-Projet Flowers, INRIA Bordeaux Sud-Ouest, 33405 Talence, France; 2 Équipe Handicap, Activité, Cognition, Santé, BPH-University of Bordeaux-Inserm (U1219), 33000 Bordeaux, France; 3 Equipe-Projet Auctus, INRIA Bordeaux Sud-Ouest, 33405 Talence, France; fage_charles@yahoo.fr; 4 Institut Polytechnique de Bordeaux (INP), 33405 Talence, France; charles.consel@bordeaux-inp.fr; 5 Centre Ressources Autisme, Centre Hospitalier Charles Perrens, 33000 Bordeaux, France; aamestoy@ch-perrens.fr (A.A.); mbouvard@ch-perrens.fr (M.B.); ketchegoyhen@ch-perrens.fr (K.E.); 6 UMR-CNRS 5293 GIN, Institut des Maladies Neurodégénératives (IMN), 33000 Bordeaux, France; isabelle.hesling@wanadoo.fr

**Keywords:** school inclusion, school adaptive behaviors, autism spectrum disorders, intellectual deficiency, cross-syndrome methodology

## Abstract

The school inclusion of students with autism is still a challenge. To address the cognitive underpinnings of school-related adaptive behaviors, 27 students with autism and 18 students with intellectual and/or severe learning disability, aged from 11 to 17, were recruited. They underwent socio-emotional processing and executive functioning assessments, as well as school-related adaptive behavior and quality of life measurements. Both groups performed equally on socio-emotional and executive assessments, and they reported the same low quality of life. However, students with autism exhibited more limitations than the students with intellectual disabilities on complex school adaptive behaviors (socialization and autonomy) and problem behaviors, but both groups performed equally on more basic adaptive behaviors (school routines, communication). Multiple regression analyses highlighted between-group differences in terms of adaptive functioning profiles, which were linked with different cognitive predictors according to students’ medical conditions. The greater school-related limitations of students with autism were mostly explained by socio-emotional performance, while IQ (intellectual quotient) mostly explained the comparable between-group limitations. The low quality of life of both groups was slightly explained by executive performance. The role of both socio-emotional and executive functioning in students’ adaptive behaviors and quality of life suggests remediation targets for promoting the school inclusion of students with autism.

## 1. Introduction

Autism spectrum disorder (ASD) is a neurodevelopmental disorder characterized by deficits in communication and social interactions, as well as restricted activities and interests (such as repetitive stereotypic behaviors) [1]. The early onset of ASD leads to limitations from a very young age in carrying out a wide range of daily activities across multiple settings. This results in restrictions on social participation for individuals with ASD, starting with school inclusion in mainstream settings. 

There is growing evidence that inclusive education may foster positive outcomes in children with ASD, improving their quality of life, academic and social development, as well as occupational future [2,3]. The Children and Youth version of International Classification of Functioning, Disability and Health (ICF-CY [4]) has identified the two domains mostly responsible for situations of school disability. The first domain is related to socio-environmental factors (e.g., a lack of accompaniment of children, insufficient teacher training about ASD, misgivings of school staff, and misunderstandings by peers) [3,5]. The second domain is related to the limited adaptive skills of children with ASD. This leads to difficulties in autonomously performing school activities that are expected in mainstream settings [6].

Few studies have addressed the relationships between school-related adaptive behaviors and the cognitive impairments associated with ASD [7]. A better understanding of the cognitive underpinnings of the adaptive and problem behaviors is critical to improve the effectiveness of psycho-educational interventions for children with ASD. Therefore, this study examined the relationships between school-related adaptive functioning and cognitive functioning in children with ASD.

### 1.1. Adaptive Functioning and Adaptive Behaviors in ASD

Adaptive functioning refers to the conceptual, social and practical skills that allow individuals to adapt to their environment and for functioning in their daily life [8]. ASD-related studies have reported that children with ASD display greater deficits in adaptive skills than children with an intellectual disability (ID) [9]. These deficits have appeared to be more profound than expected regarding their intellectual level and have suggested that ASD has consequences on adaptive functioning (e.g., [10,11,12]). Deficits in adaptive skills are linked to problem behaviors (also termed maladaptive or challenging behaviors in some studies), such as stereotypies, self-injury, aggression, and tantrums [13]. The assessment of both cognitive level and adaptive functioning is highly recommended for determining the amount of daily life limitations that occur in ASD [14]. 

Measurements of adaptive behaviors are often parent- or teacher-reported measures, and they concern the occurrence of a large set of behaviors distributed across several dimensions (e.g., socialization, communication, and daily living skills). Several evaluation tools have been developed to assess adaptive behaviors, such as the Vineland Adaptive Behavior Scale (VABS, [15]) or the “Echelle Québécoise des Comportements Adaptatifs—Version Scolaire” (EQCA-VS, [16]). Children with ASD typically display a particular profile in the measurement of adaptive behaviors, with greater impairments in socialization areas and intermediate deficits in communication areas (e.g., [10,11,17]). 

Improvements of adaptive functioning have already been reported to foster more positive outcomes in adulthood than improvement at the cognitive level [10]. Moreover, some studies have stated that quality of life (QoL) is positively related to better adaptive behavior scores and negatively correlated with other autistic symptoms scales [18]. This further supports the importance of studying adaptive functioning in people with ASD and their relationship with their cognitive profile.

### 1.2. Adaptive Behaviors and General Factors (Age, IQ and ASD Severity)

Positive associations between IQ (intellectual quotient) and overall adaptive skills in children with ASD suggest that general intellectual functioning is globally linked to adaptive behaviors [10,14,17]. However, some studies have reported that IQ levels do not always predict adaptive behaviors and social functioning in ASD [9,19]. Adaptive skills in ASD appear to be more impaired than expected regarding their intellectual functioning [10,11]. These results suggest that intellectual functioning is unlikely to be the only cognitive factor related to adaptive functioning. 

The relationships between adaptive functioning and ASD symptom severity are still a matter of debate because of inconsistent findings. Strong negative correlations [19,20] and weak associations [9,11] between ASD symptom severity and adaptive behaviors have been reported across studies.

A recent study showed that high intellectual functioning and less ASD symptom severity are associated with greater adaptive functioning for young children but not for older children with ASD [8]. Overall, these data pinpoint the complex and possible interactive loop between cognitive functions, adaptive behaviors, and child development. 

### 1.3. Adaptive Behaviors and Specific Cognitive Factors (SEP and Executive Functions)

Some studies have focused on the relationships between adaptive functioning and socio-emotional processing (SEP) in ASD, including Theory of Mind (ToM) deficits. A study reported that ASD severity measures (such as SRS: Social Responsiveness Scale) are associated with ToM abilities [20]. In children with ASD without ID (i.e., with an IQ > 70), the facial recognition of sadness appears to be correlated with ASD symptoms and adaptive behaviors [21]. In this study, authors also showed that the facial recognition of basic emotions is correlated with adaptive behavior occurrence.

Executive functions (EF) have also been documented in regard to socio-communicative and behavioral impairments. Subjects with ASD exhibit difficulties in EF [22] such as joint attention, cognitive flexibility, self-monitoring, executive control, initiation, planning and inhibition [23,24]. Impairments in EF lead to several daily difficulties, notably in evaluating and responding to social situations and adapting their behavior, thus possibly compromising the success of school inclusion [25]. However, few studies on individuals with ASD have explored the links between executive functions and adaptive behavior. The authors of [26] reported opposite EF profiles in children with ASD, depending on its association with an ID. Children with ASD appear to be more impaired in planning, set shifting, and behavioral EF, whereas children with ASD and ID are more impaired in inhibition and generativity. Both profiles have been related to poorer adaptive abilities, especially on socialization. The authors of [27] showed that EF problems explain 12.3% of the variance in daily living skills, 13% of the variance in socialization skills, but were not contributive in communication skills. These authors also showed that metacognition and behavioral regulation problems explain part of socialization skills (16.8%) and communication skills (5%). Working memory problems have been more linked to communication and daily living skills. Inhibition performance has been found to predict global adaptive scores [27]. 

In summary, the relationships between cognitive functions and adaptive functioning in ASD appear to be complex and remain unclear because of inconsistent findings [10]. Evidence from studies in this field suggests that the adaptive behaviors in children with ASD are mediated by both general factors (IQ and ASD severity) and specific cognitive factors (socio-emotional and executive attentional processes). All these factors deserve consideration for a better understanding of the adaptive behaviors of children with ASD in school settings. 

However, very few studies have simultaneously investigated effects of several cognitive factors on adaptive functioning in children with ASD (e.g., [11]). The aim of this work was then to investigate the relationships between the general factors (e.g., IQ and age), the specific cognitive mediators (socio-emotional processing and executive functions), and the socio-adaptive limitations that occur in students with ASD aged from 11 to 17 years old.

To better assess the role of intellectual functioning, a control group with students without ASD but presenting either learning impairments or an ID according to the cross-syndrome method was enrolled [28]. This design allowed for the depiction of a differential profile for children with ASD and for the identification of the specific influence of ASD on socio-adaptive functioning.

## 2. Materials and Methods

The ethics committee of the university of Bordeaux approved the experiment protocol before the participants’ recruitment. Both parental informed consent and students’ assent were collected according to the Helsinki convention.

### 2.1. Participants

Participants were secondary school students from special education classrooms who were included in a mainstream environment at least one hour per week. A total of 50 students between 11 and 17 years old were initially included in our study. Five of them were excluded from our analysis because of a large amount of missing values due to testing difficulties (barriers in verbal communication). Finally, 45 students were enrolled in either the experimental or the control group according to their diagnostic.

Child psychiatrists from a pediatric neurology service diagnosed all the students. The ASD diagnosis was performed according to DSM-5 criteria (Diagnostical and Statistical Manual – 5^th^ version [1]) for ASD and their results for ADI-R (Autism Diagnostic Interview-Revised [29]) and ADOS (Autism Diagnostic Observation Schedule [30]). A first group (referred as the ASD group) was composed with 27 students with ASD with or without ID, and a second group (referred as the ID group) was composed of 18 students—15 were students with mild-to-severe ID, and 3 were students with a severe learning disability. ID is characterized by cognitive, social and adaptive impairments, and it is notably determined by the IQ score (<70, according to Wechsler Intelligence Scale for Children-Fourth Edition (WISC-IV) [31]). This deficiency is generally associated with symptoms close to ASD such as stereotypies and problem behaviors [14]. 

The two groups were matched by chronological age (*t* (43) = 0.205; *p* > 0.800) and by intellectual functioning (*t* (43) = 1.347; *p* > 0.100), which was estimated from the abbreviated version of WISC-IV [32]. Comparison tests between these two groups were performed with parametric analyses because data were normally distributed; results and means are reported in Table 1. 

Prior to the experiment, we collected both parental informed consent and students’ assent, according to the Helsinki convention. Moreover, the ethics committee of the university approved the experiment protocol before the participants’ recruitment.

### 2.2. Measures

To investigate the relationships between cognitive functioning and socio-adaptive functioning, a set of questionnaires and neuropsychological tests were administered (see Appendix A for a detailed description of tests). Questionnaires were administered at school (for students and teachers) or at home (for parents). 

#### 2.2.1. Cognitive Functioning Assessment

Two main domains were assessed: (1) socio-cognitive functioning and social skills; and (2) executive functioning. The set of tests was administered in a randomized order, in three sessions of 1 h.

As for global cognitive processing, socio-emotional processing can be divided between low-level, automatic, inflexible, and quick processes (such as emotion identification and face recognition) and high-level, flexible, effortful and slow processes (such as intention attribution and emotional awareness) [33,34]. Hence, to assess low- and high-level of socio-emotional functioning, a set of eight tests from ASD literature was selected as follows:

- Low-level socio-emotional functioning was assessed with an emotion identification from static faces test [35], an emotion identification from gazes test [36], an emotion identification from dynamic faces test [37], and the faces memory subtest from NEPSY (Developmental Neuropsychological Assessment [38]).
- High-level socio-emotional functioning was assessed with the LEAS-C (Level of Emotional Awareness Scale for Children [39]), the emotional fluency test [40], the PEPS-C (Profiling Elements of Prosodic Speech for Children in its French version [41]), and the intention attribution test [42].

To assess executive attentional functioning, a set of subtests was selected from the French version of the TAP battery (Test for Attentional Performance [43]): working memory, divided attention, set shifting, and go/no-go subtests.

#### 2.2.2. Assessment of Socio-Adaptive Behaviors and Social Skills

The socio-adaptive functioning and social skills were assessed with two hetero-reported questionnaires (see details in Appendix B): the EQCA-VS [16] and the SRS-2 [44]. These were both filled out by the parents and the specialized teacher. Then, we computed the average scores from parents and specialized teacher scores to obtain unique measures per individual. This ensured a more objective measure, because some studies have shown discrepancies between parents’ and teachers’ ratings for adaptive and social skills assessment [45]. This is particularly true when the children’s disability has been identified [46]. Inter-rater reliability was assessed using intra-class correlations (ICC), and showed a good-to-excellent inter-rater agreement (0.40 < ICC < 0.80), except for the maladaptive behaviors dimension (ICC < 0.20).

- The EQCA-VS [16] is a francophone parent- and/or teacher-reported assessment of adaptive behaviors at school. Adaptive behaviors are divided into five dimensions: (1) communication, (2) socialization, (3) autonomy, (4) preschool and school abilities and (5) leisure; maladaptive behaviors refer to violent behaviors, withdrawal behaviors, unacceptable behaviors and habits, antisocial behaviors and inadequate sexual behaviors (higher scores revealed the greater occurrence of adaptive or problem behaviors).
- The SRS-2 [44] is a parent- and/or teacher-reported 65-items questionnaire designed to quantitatively measure the ability of a child to engage emotionally in reciprocal social interactions in naturalistic social settings. Five areas of reciprocal social interactions are rated: (1) social awareness; (2) social cognition; (3) social communication; (4) social motivation; and (5) autistic mannerisms including stereotypies, restricted interests, and repetitive behaviors. The higher the score, the more severe the social impairments, and an R-score higher than 60 indicates a high probability of ASD. The SRS was used as a measure of (mal)-adaptive behaviors in social settings more than a measure of ASD severity. Indeed, the SRS score has been revealed to be influenced by non-ASD-related features such as age or cognitive level [47].

#### 2.2.3. Quality of Life (QoL) Measure

For assessing the quality of the school life, students filled out the AuQuEI (Autoquestionnaire Qualité de vie Enfant Imagé). This is an auto-questionnaire about the quality of life including items closely related to school life [48]. The AuQuEI includes an open question test about pleasant and unpleasant situations experienced by the child and a closed-question scale with 33 items on several areas of daily life. The child answers each item with a pictorial scale, from “not happy at all” with a sad face to “very happy” with a happy face. The score is an overall percentage of satisfaction.

### 2.3. Statistical Analyses

Statistical analyses were computed with R software version 3.2.3. 

All of our measures were normally distributed. Between-group comparisons were conducted with parametric Student *t*-tests for independent samples on all measures. A sensitivity analysis was performed to determine the minimal effect size that could be detected with our sample. This analysis was performed with G*Power 3.1 software [49]. For a power of 80%, the minimal effect size that could be detected with our study was η^2^ = 0.160.

In preparation of multiple regression analyses, we computed a parametric Pearson correlation matrix to identify strong correlations between the measures of our set. We used significantly correlated measures to compute composite scores from socio-emotional processing (SEP) and executive-attentional functioning (EaF) measures. 

Finally, multiple regression analyses were performed on socio-adaptive and QoL measures. Once IQ and age were entered, we ran stepwise multiple regression analyses and kept variables that significantly predicted the dependent measure. Regression analyses were firstly performed on the whole group. When the pathology significantly contributed to the model, we ran regression analyses separately for each group condition (students with ASD and students with ID).

For any significant model, we computed the contribution of each significant predictor to the explained variance, with a hierarchical variance partitioning algorithm [50]. This latter consisted of computing parts of explained variance that are only due to the effect of the considered independent variable or due to the combined effect of two or more predictors.

## 3. Results

### 3.1. Neuropsychological Results

Student *t*-tests failed to reach a significant level for all the collected measures (see Table 2 for details). Consequently, there were no significant differences between students with ASD and students with ID irrespective of measures (socio-emotional or executive attentional measures).

### 3.2. Construction of Cognitive Mediators

The correlation matrix was computed with a Bonferroni correction for multiple comparisons, and it yielded two inter-correlated sets of variables (see Table 3). The first component was composed of all socio-emotional measures except the dynamic emotion identification and the LEAS. The second group was composed of three TAP subtests: go/no-go, divided attention and set shifting. 

Composite measures were computed by averaging students’ z-scores on each contributive measure. The two built composite measures were named from the included measures: SEP (socio-emotional processing) and EaF (executive-attentional functioning) factors. As for neuropsychological results, students with ASD and students with ID did not significantly differ on either the SEP factor (*t* (43) = 0.234; *p* > 0.800) or on the EaF factor (*t* (43) = 0.875; *p* > 0.300) (Table 2).

### 3.3. Group Differences on Socio-Adaptive Functioning and on QoL

For EQCA-VS measures, Student *t*-tests revealed significant differences for two dimensions of adaptive behaviors (Table 4). Students with ASD had significantly lower scores in socialization (t (43) = −3.536; *p* < 0.002; η^2^ = 0.221) and autonomy (t (43) = −2.964; *p* < 0.006; η^2^ = 0.166) than those of students with ID. On the other dimensions, the maladaptive total score was also significantly different between the two groups with a relatively large effect (t (43) = 2.514; *p* < 0.01; η^2^ = 0.126). Students with ASD exhibited more problem behaviors than students with ID. 

Concerning the SRS scores, Student *t*-tests revealed significant differences between students with ASD and students with ID for the total score (*t* (43) = 3.658; *p* < 0.01; η^2^ = 0.233) with a large size effect (Table 4). Students with ASD displayed higher socio-adaptive impairment than students with ID. 

On the AuQuEI total satisfaction score, Student *t*-tests revealed no significant differences between the two group conditions (*t* (43) = −0.196; *p* > 0.800), with around 62% of satisfaction for both groups (Table 4). 

### 3.4. Mediating Effects on Socio-Adaptive Functioning and QoL

A set of multiple regression analyses was performed on each dependent measure (EQCA-VS, SRS and AuQuEI scores) following this procedure. First, ascendant regression analyses were performed on the whole group with three entered predictors (pathology: nominal variable distinguishing ASD and ID conditions; the SEP and EaF factors). Second, when pathology was significantly contributive to the best model, group-separated regression analyses were carried out with the SEP and EaF factors as predictors. For all the regression analyses, the IQ and age variables were entered in the model for controlling their possible effects. The regression details are documented for each EQCA-VS subscore, for the SRS total score, and for the AUQUEI score in Table 5 for the whole group, in Table 6 for the regression analyses performed on the ASD group, and in Table 7 for those performed on the ID group.

#### 3.4.1. Regression Results for EQCA-VS Subscores

Socialization: A regression analysis on the whole group revealed a significant model that accounted for 28.5% of the variance. The socialization score was predicted by pathology (22.2% of the variance) and by IQ (5.7% of the variance). Group-separated regression analyses for the ASD group revealed a significant model with 24.5% of explained variance including only the SEP factor (17.2% of the explained variance). In the ID group, the best significant model accounted for 68.4% of the variance, with three significant predictors: IQ (26.6% of the variance), age (25.5% of the variance) and the SEP factor (16.3% of the variance). 

Autonomy: A regression analysis on the whole group revealed a significant model that accounted for 27.6% of the variance. The autonomy score was predicted by pathology (17.6% of the variance) and by IQ (9.5% of the variance). Group-separated regression analyses for the ASD group revealed a significant model with 43.6% of explained variance including only the SEP factor (28% of the explained variance). In the ID group, the best significant model reached 60.1% of the variance, with three significant predictors: IQ (22.7% of the variance), age (19.5% of the variance) and the SEP factor (17.9% of the variance). 

Communication: The best significant model accounted for 20.3% of the variance with IQ as a unique predictor (20.2% of the variance). 

School Routines: The best model accounted for 29.2% of the variance with IQ as a unique predictor (29% of the variance). 

Leisure: No model reached the significance level with the studied predictors.

Maladaptive behaviors: A regression analysis on the whole group revealed a significant model that accounted for 26.5% of the explained variance. The maladaptive behaviors score was predicted by pathology (12% of the variance). Group-separated regression analyses for the ASD group revealed a significant model, reaching 25.8% of variance with the SEP factor as a unique predictor (16.5% of the variance). In the ID group, the significant model reached 43.9% of the variance, with two significant predictors: IQ (19.6% of the variance) and age (24.2% of the variance).

#### 3.4.2. Regression Results for SRS Score

A regression analysis on the whole group revealed a significant model accounting for 25.5% of the variance and only included pathology as a variable (22.1% of the variance). Group-separated regression analyses for the ASD group revealed a significant model with 23.5% of explained variance and with the SEP factor as a unique predictor (17.3% of the variance). In the ID group, the best significant model accounted for 45.7% of the variance, with three significant predictors: IQ (20.9% of the variance), age (10.3% of the variance) and the SEP factor (6.8% of the variance).

#### 3.4.3. Regression Results for AuQuEI Score

The whole-group regression analysis revealed a significant model accounting for 16.2% of the variance with the EaF factor as a unique predictor (9.9% of the variance). 

## 4. Discussion

The purpose of our study was to simultaneously investigate the relationships between general factors (i.e., age and IQ), specific cognitive mediators (i.e., socio-emotional and executive functioning) and school-related socio-adaptive limitations that occur in students with ASD, as compared to student with ID. First of all, the similarities and differences between the ASD and ID conditions have been investigated for socio-emotional and executive functioning as well as for adaptive behaviors and QoL measures.

### 4.1. Similarities and Differences between ASD and ID Conditions

Students with ASD and those with ID have exhibited nearly equal socio-emotional and executive functioning. Deficits in socio-emotional functioning have been widely documented for children with ASD [50] and at a lesser extent for children with ID [51,52]. Additionally, the authors of [53] reported lower face recognition performances in children with ASD or with Down syndrome than in typically developing children. Similarly, an impaired executive functioning has been reported for both ASD and ID children, compared with typically developing children (e.g., [24,26,54,55]). Finally, in a study with a sample-based design very close to ours, the authors of [56] found that performances on shifting, inhibition and updating in young adults with ID and with/without comorbid ASD were not significantly different.

Socio-adaptive measures were lower in students with ASD than in students with ID for socialization and autonomy behaviors, as well as for maladaptive behaviors scores from the EQCA-VS. Similarly, as a hallmark of ASD condition, ASD students obtained SRS scores (m = 89.40) that exceeded the clinical cutoff (R-scores >60) and that were largely higher than those obtained for students with ID (m = 57.53). These results are consistent with previous studies, which respectively investigated the two medical conditions (e.g., [10,11,12,27,52]). The ASD vs. ID comparison has revealed, for the first time, a greater socio-adaptive profile in students with ID as compared to those with ASD. More particularly, our results emphasize the behavioral complexity in which communication skills such as school routines are more basic adaptive behaviors than socialization or autonomy behaviors [10,26].

Third, no significant group differences were observed in the AUQUEI score (QoL). This fits with results from [57], which revealed that both children with ASD and children with ID reported a similarly low QoL. 

From the overall data, the present students with ASD and those with ID shared similarities in terms of socio-emotional and executive functioning while they were distinguished by limitation differences in adaptive behaviors, with a greater limitation in the ASD condition. Despite of this, both groups reported an equally low QoL.

### 4.2. Mediating Effects of Studied Factors on School Adaptive Behaviors in Students with ASD or ID

As a result of group-related differences previously observed, the multiple regression analyses on EQCA-VS measures for the whole group revealed that the pathology variable elicited a significant mediating effect for socialization (22.2%), autonomy (17.6%) and maladaptive behaviors (9.2%) scores, as well as for the SRS total score (22.1%). The significant contribution of pathology condition emphasizes the greater impairments in socio-adaptive behaviors in students with ASD than in students with ID. 

These four scores were all significantly explained by the SEP factor in students with ASD, with a contribution comprised between 16.5% and 28% (Table 6). By contrast, these scores elicited different significant models in the ID group: the combination of IQ, age and SEP factors produced between 43.9% and 68.4% of explained variance. In these models, IQ and age had the largest contributions to the models (10.3%–26.60%), whereas the SEP factor was less (6.8%–17.9%) to not contributive (Table 7).

The present results suggest that the specific SEP factor is a critical factor for explaining the highly limited socio-adaptive behaviors (i.e., autonomy, socialization, maladaptive behaviors and SRS score) in students with ASD. For ID condition, it is noteworthy that IQ and age are the best mediators of almost all studied socio-adaptive dimensions as reported in numerous ID studies [52,58].

For other dimensions of EQCA-VS, only IQ elicited a large part of explained variance for both communication (20.2%) and school routines (29.0%) scores of the whole of participants. Furthermore, IQ brought additional contribution in models for socialization, autonomy and maladaptive behaviors. Thus, the present results reinforce the critical role played by the general intellectual functioning in adaptive behaviors widely demonstrated in the ID condition [59], in ASD condition [8,10] or even in typically developing children [26,60].

From the overall results, it can be seen autonomy and socialization scores were less dependent on IQ than communication and school routines, while they were sensitive to the pathology condition with the specific SEP factor as the best predictor for the ASD condition and the general factors (i.e., IQ and age) as the best predictors for the ID condition. Such a conclusion echoes ASD studies that have raised the question of the relevance of IQ value in children with ASD [19].

The EaF factor failed to bring a significant mediating effect of all measures of school adaptive behaviors (EQCA-VS score) and social skills (SRS scores) in both the ASD and ID conditions. However, executive impairments were actually observed in both groups. These results are not consistent with the core of studies that have highlighted the critical role of executive impairments in reduced adaptive behaviors in children with ASD or with ID [26,27,60]. These inconsistencies could be explained in various ways. 

First, previous studies have investigated the EF factor alone without the competition of the ToM factor [26,27]. Hence, the role of the EaF factor could be artificially inflated compared to our study where the EaF and SEP factors are competitors in regression analyses. Second, another explanation could be related to discrepancies of EaF assessment across the present study and previous ones. This explanation is notably informed with the task complexity effect and the hetero-rating biases in the literature of executive dysfunction in autism (e.g., for reviews [23,25]). In the present study, EaF was assessed with objective measures in computerized attentional tasks (TAP tests) for both reducing the social component and measuring almost selectively single executive processes such as cognitive flexibility (i.e., set shifting test), inhibition (i.e., the go/no-go test), and divided attention (i.e., the divided attention test). By contrast, most previous studies have used either objective measures of EF based on complex tasks [26,56] or on subjective rating scales [24,60]. However, several authors have claimed that the task complexity can confuse the interpretation of results since complex executive tasks like the WCST (Wisconsin Card Sorting Test [61]) or the Hanoi/London Tower test [62] require multi-component processes [63]. Additionally, subjective EF measures such as the BRIEF questionnaire (Behavior Rating Inventory of executive functions [64]) are often used, although some ASD studies have revealed mismatches between subjective EF measures and actual EF measures [24,60].

To sum up, limited complex socio-adaptive behaviors (autonomy, socialization and social skills) and problem behaviors at school have been mostly explained by the SEP factor in students with ASD, while the more basic adaptive behaviors (communication and school routine) have been explained by IQ. By contrast, for students with ID, all the aspects of adaptive behaviors at school have been greatly explained by IQ and age, even if the SEP factor modestly contributed to complex adaptive behaviors (autonomy and socialization).

### 4.3. Mediating Effects of Studied Factors on QoL in Students with ASD or ID

Regarding QoL, scores from AUQUEI were significantly explained by the EaF factor (9.9%). The EaF effect on QoL of students with ASD was also reported in [55]. However, these authors have reported that EF (probed with the BRIEF test) predicted 66% of accounted variance of the QoL score (measured with PedsQL [Pediatric Quality of Life Inventory]) in children with ASD [55]. Once again, the above explanations could explain the size difference in the effect of EF on QoL. Be that as it may, the EF factor appears to be a stable contributor of QoL in students with ASD as well as in students with ID. In line with previous studies that included children with an average IQ of above 70 [55], we did not find that IQ was related to QoL.

The association between EaF and QoL could be accounted for by the relation between QoL and decision-making processes. Indeed, EFs are well recognized as playing a critical role in decision-making processes [65]. Additionally, several studies have reported close links between QoL in children and their capabilities of decision-making. Thus, as scaffolding, EFs could contribute to decision-making processes, which in turn could influence the QoL of students with ASD or with ID.

### 4.4. Limitations

The main limitation of our study comes from our relatively small sample, which restricted the statistical power of reported results, as well as the number of studied cognitive mediators. Indeed, the amount of cognitive accounts for ASD is larger than that we investigated; for instance, the enhanced perception account [66] that stresses the role played by hyper- or hypo- visual and auditory processing in ASD or even the weak central coherence account [67] that emphasizes processing distortions in local vs. global information, and so on (for review, [68]). In the same vein, the inclusion of other frequent ASD-related impairments, such as emotional dysregulation, motor dysfunctions, sensory hyper/hypo-sensitivity, or language impairments would be informative. Taken together, all these accounts increase the likelihood of successfully drawing out a cognitive phenotype of students with ASD relative to school adaptive behaviors. 

Another limitation is related to inter-individual variability within the ASD group, notably with regard to IQ. However, in our study, the recruitment took place in a specialized classroom, where students may have more severe cognitive disabilities than students included in mainstream classrooms. Furthermore, the adding of a sample consisting of typically developed students could probably give more insights to contrast the ASD and ID conditions.

## 5. Conclusions

In summary, both students with ASD and those with ID showed impairments in socio-emotional and executive attentional functioning, although they exhibited different patterns of adaptive behaviors. There were qualitative differences between ASD and ID students in the cognitive underpinnings of adaptive functioning. Indeed, while both general factors (i.e., age and IQ) and the SEP factor were found to mediate the quality of adaptive behaviors in ID students, only the SEP factor appears to be critical in ASD students’ adaptive functioning. Moreover, both groups exhibited a low QoL.

Our study was a first attempt to highlight the single effect and combined influences of general and specific factors that may influence the school handicap situation of students with ASD. Further studies should investigate a larger range of cognitive mediators according to recent cognitive accounts to draw out a more complete cognitive phenotype of students with ASD. A larger sample size, which would consider the entire autistic spectrum and comprise typically developing children, may also highlight the strengths and weaknesses of students with ASD to build the cognitive phenotype and broaden the set of possible remediation’s targets.

## Figures and Tables

**Table 1:** Demographic data (N (number), age, IQ (intellectual quotient)).

	All	ASD	ID	Between-Group Comparisons		
				*t*	df	*p*
N	45	27	18			
Age	14.23 (1.35)	14.26 (1.39)	14.17 (1.32)	0.234	43	0.816
IQ	69.60 (26.30)	74.00 (30.10)	63.00 (18.13)	1.389	43	0.172

***Note***. df: degree of freedom; IQ = intellectual quotient; ASD = autism spectrum disorder; ID = intellectual deficiency; SD = standard deviation.

**Table 2:** Means, SD and *t*-test for cognitive and executive assessment.

	ASD	ID	Between-Group Comparisons				
			*t*	*ddl*	*p*	*η^2^*	β *^1^*
Working Memory	11.48 (3.43)	13.22 (2.84)	−1.781	43	0.082	0.067	0.573
Divided Attention	725.26 (174.8)	800.95 (112.76)	−1.622	43	0.112	0.056	0.620
Set Shifting	1109.11 (459.11)	970.82 (315.51)	1.232	43	0.225	0.033	0.796
Go/No-Go	444.03 (86.11)	476.77 (75.06)	−1.553	43	0.128	0.052	0.744
Face Memory	9.87 (3.34)	11.47 (3.25)	−1.592	43	0.119	0.054	0.659
Facial Emotion ID˚	19.85 (5.76)	19.61 (5.29)	0.142	43	0.888	0.000	0.948
Gaze Emotion ID˚	8.67 (2.53)	7.06 (3.44)	1.812	43	0.077	0.069	0.597
Dynamic Emotion ID˚	5.85 (1.87)	5.22 (2.18)	−1.203	43	0.236	0.032	0.831
Intention Attribution	8.89 (3.85)	8.50 (3.58)	0.341	43	0.735	0.003	0.937
Emotional Fluency	4.81 (2.73)	4.67 (2.17)	0.193	43	0.848	0.001	0.946
LEAS-C	17.18 (5.49)	19.00 (4.45)	−1.169	43	0.249	0.030	0.784
PEPS	25.99 (5.70)	26.22 (7.70)	−0.145	43	0.885	0.000	0.949
SEP Factor	13.61 (2.98)	13.79 (2.67)	−0.206	43	0.838	0.001	0.945
EAF Factor	749.73 (216.21)	749.51 (136.07)	0.004	43	0.997	0.000	0.995

***Note****. ASD* = Autism Spectrum Disorder; *ID* = Intellectual Deficiency; ID˚ = Identification; *LEAS-C* = Levels of Emotional Awareness Scale for Children; *PEPS* = Profiling Elements of Prosodic Speech for Children; SEP = Socio-Emotional Processing; *EaF* = Executive attentional Functions; *SD* = Standard Deviation ***^1^*** Type II error probability obtained from post-hoc power analysis (power = 1−β).

**Table 3:** Correlation matrix.

	WM	DA	SS	GNG	FM	FEI	GEI	IA	Flu.	LEAS	PEPS	DEI
WM	-	0.280	0.016	−0.048	0.585 *	0.369	0.172	0.233	0.376	0.301	0.314	0.232
DA		-	0.468 *	0.334 *	0.230	0.418 *	0.082	0.048	0.167	−0.036	0.141	−0.122
SS			-	0.250	−0.009	0.279	0.074	−0.109	−0.035	−0.122	−0.156	−0.155
GNG				-	−0.026	−0.091	−0.097	−0.237	−0.156	−0.140	−0.140	0.109
FM					-	0.608 *	0.427 *	0.471 *	0.341	0.228	0.423 *	0.420 *
FEI						-	0.522 *	0.542 *	0.504 *	0.127	0.352	0.258
GEI							-	0.471 *	0.226	0.130	0.278	0.335
IA								-	0.484 *	0.149	0.240	0.358
Flu.									-	0.307	0.405	0.298
LEAS										-	0.261	0.131
PEPS											-	0.152
DEI												-

**Note**. *p < 0.05 (with Bonferroni’s correction). WM: working memory; DA: divided attentions; SS: set shifting; GNG: go/no-go; FM: face memory; FEI: facial emotion identification; GEI: gaze emotion identification; IA: intentions attributions; Fiu: fluence; DEI: dynamic emotion identification.

**Table 4:** Means, SD (standard deviation) and *t*-tests for socio-adaptive and quality of life (QoL) measures.

		*ASD*	*ID*	Between-Group Comparisons				
				*t*	*ddl*	*p*	*η^2^*	β *^1^*
EQCA-VS	Communication	25.24 (7.83)	27.00 (6.06)	−0.806	43	0.424	0.015	0.873
	Socialization	20.69 (8.00)	28.36 (5.52)	−3.536	43	0.001	0.221	0.052
	Autonomy	22.60 (6.76)	28.32 (5.64)	−2.964	43	0.005	0.166	0.161
	School Routines	38.77 (7.75)	37.56 (8.73)	0.490	43	0.627	0.005	0.924
	Leisure	22.27 (6.75)	22.08 (9.35)	0.076	43	0.940	0.000	0.949
	Maladaptive	19.48 (15.09)	9.82 (7.40)	2.514	43	0.007	0.126	0.258
SRS	Total score	89.41 (30.02)	57.53 (26.38)	3.658	43	0.006	0.233	0.048
AUQUEI	Total	61.77 (13.04)	62.78 (11.39)	−0.196	43	0.846	0.001	0.942

***Note***. ASD = autism spectrum disorder; *ID* = intellectual deficiency; EQCA-VS = Echelle Québécoise des Comportements Adaptatifs—Version Scolaire; SRS = Social Responsiveness Scale; AUQUEI = Autoquestionnaire Qualité de Vie Enfant Imagé; SD = standard deviation. *^1^* Type II error coefficient obtained from post-hoc power analysis (power = 1−β).

**Table 5:** Multiple regression analyses on socio-adaptive and QoL measures in the whole group.

Predicted Measures		Mod	R^2^	Adj. R^2^	ΔR^2^	F	Cst	Pred.	β	Contrib.
EQCA-VS	Communication	1	0.202	0.164	-	5.326 **	0.000	*IQ*	0.450 **	0.160
								*Age*	0.008	0.000
	Socialization	1	0.042	−0.003	-	0.928	0.000	*IQ*	0.184	0.035
								*Age*	−0.083	0.008
		2	0.286	0.234	0.244	5.479 **	−0.412 *	*IQ*	0.287 *	0.047
								*Age*	−0.061	0.005
								*Pathology*	0.505 ***	0.182
	Autonomy	1	0.075	0.031	-	1.712	0.000	*IQ*	0.259	0.028
								*Age*	−0.077	0.003
		2	0.276	0.223	0.201	5.212 **	−0.373 *	*IQ*	0.353 *	0.076
								*Age*	−0.057	0.005
								*Pathology*	0.458 **	0.142
	School Routines	1	0.292	0.259	-	8.669 ***	0.000	*IQ*	0.541 ***	0.257
								*Age*	0.053	0.001
	Leisure	0	0.088	0.044	-	2.016	0.000	*IQ*	0.276	0.039
								*Age*	−0.092	0.005
	Maladaptive	1	0.008	−0.039	-	0.169	0.001	*IQ*	−0.086	0.007
	Behaviors							*Age*	−0.027	0.001
		2	0.152	0.089	0.144	4.074 *	0.001	*IQ*	0.420 *	0.017
								*Age*	0.021	0.000
								*SEP*	−0.691 **	0.072
		3	0.298	0.227	0.146	4.235 **	0.227	*IQ*	0.285	0.024
								*Age*	0.001	0.000
								*SEP*	−0.583 **	0.121
								*Pathology*	−0.277 *	0.082
SRS	Total Score	1	0.019	−0.028	-	0.402	−0.001	*IQ*	−0.125	0.016
								*Age*	0.050	0.003
		2	0.255	0.201	0.236	4.680 **	0.405 *	*IQ*	−0.226	0.026
								*Age*	0.028	0.002
								*Pathology*	0.497 ***	0.173
AUQUEI	Total Score	1	0.071	0.027	-	1.616	0.000	*IQ*	−0.238	0.021
								*Age*	−0.135	0.006
		2	0.144	0.081	0.073	2.301 *	0.000	*IQ*	−0.218	0.028
								*Age*	−0.135	0.009
								*EaF*	0.270 *	0.044

**Note**. *p < 0.05. **p < 0.01. ***p < 0.001. Mod = model, Adj. R^2^ = adjusted R^2^; ΔR^2^ = difference in R^2^ compared with previous model; Cst = constant; Pred. = predictor; Contrib. = contribution of the single variable.

**Table 6:** Multiple regression analyses on socio-adaptive and QoL measures in the Autism spectrum disorder (ASD) group.

Predicted Measures		Mod.	R^2^	Adj. R^2^	ΔR^2^	F	Cst	Pred.	β	Contrib.
EQCA-VS	Socialization	1	0.126	0.053	-	1.727	−0.422	*IQ*	0.362	0.050
								*Age*	0.117	0.003
		2	0.321	0.232	0.195	3.620 *	−0.295	*IQ*	−0.346	0.054
								*Age*	−0.061	0.003
								*SEP*	0.820 *	0.176
	Autonomy	1	0.213	0.148	-	3.252	−0.387 *	*IQ*	0.472 *	0.140
								*Age*	0.144	0.007
		2	0.510	0.446	0.297	7.970 ***	−0.234	*IQ*	−0.401	0.106
								*Age*	−0.075	0.005
								*SEP*	1.011 **	0.335
	Maladaptive	1	0.063	−0.015	-	0.804	0.325	*IQ*	−0.185	0.026
	behaviors							*Age*	−0.215	0.037
		2	0.282	0.188	0.219	3.008 *	0.174	*IQ*	0.566	0.040
								*Age*	−0.027	0.015
								*SEP*	−0.869 *	0.133
SRS	Total Score	1	0.064	−0.013	-	0.827	0.405	*IQ*	−0.256	0.058
								*Age*	−0.105	0.006
		2	0.241	0.141	0.176	2.427 *	0.293	*IQ*	0.417	0.031
								*Age*	0.063	0.002
								*SEP*	−0.779 *	0.108

***Note.*** *p < 0.05. **p < 0.01. ***p < 0.001. Mod = model, Adj. R^2^ = adjusted R^2^; ΔR^2^ = difference in R^2^ compared with previous model; *Cst* = constant; Pred. = predictor; Contrib. = contribution of the single variable.

**Table 7:** Multiple regression analyses on socio-adaptive and QoL measures in the intellectual disability (ID) group.

Predicted Measures		Mod	R^2^	Adj R^2^	ΔR^2^	F	Cst	Pred.	β	Contrib.
EQCA-VS	Socialization	1	0.320	0.229	-	3.521	0.650 ***	*IQ*	0.454	0.140
								*Age*	−0.538 *	0.088
		2	0.634	0.556	0.315	8.084 **	0.796 ***	*IQ*	0.957 ***	0.214
								*Age*	−0.691 **	0.212
								*SEP*	−0.732 **	0.130
	Autonomy	1	0.228	0.125	-	2.217	0.575 *	*IQ*	0.378	0.047
								*Age*	−0.459	0.079
		2	0.550	0.454	0.322	5.702 **	0.749 ***	*IQ*	0.887 **	0.167
								*Age*	−0.613 **	0.152
								*SEP*	−0.740 **	0.135
	Maladaptive	1	0.440	0.365	-	5.881 *	−0.530 ***	*IQ*	−0.562 *	0.196
	behaviors							*Age*	0.608 *	0.243
SRS	Total Score	1	0.229	0.126	-	2.221	−0.679 **	*IQ*	−0.456	0.141
								*Age*	0.384	0.088
		2	0.439	0.319	0.211	3.658 *	−0.821 ***	*IQ*	−0.868 **	0.166
								*Age*	0.509 *	0.084
								*SEP*	0.599 *	0.069

***Note.*** *p < 0.05. **p < 0.01. ***p < 0.001. Mod = model, Adj. R^2^ = adjusted R^2^; ΔR^2^ = difference in R^2^ compared with previous model; *Cst* = constant; Pred. = predictor; Contrib. = contribution of the single variable.

## Appendix A. Detailed Description of Scales

To assess low- and high-level of socio-emotional functioning, a set of eight tests from ASD literature was selected as follows

Low-level socio-emotional functioning was assessed with an emotion identification from static faces test, an emotion identification from gazes test, an emotion identification from dynamic faces test, and the faces memory subtest from NEPSY.

The emotion identification from static faces test (Ekman, 1972) consisted of presenting pictures of faces and asking the student to identify the emotion of the observed character. Thirty pictures of faces with a basic emotion were pseudo-randomly presented. This test was scored in terms of the number of correct answers.

In the emotion identification from gazes test (Baron-Cohen et al., 2001), the student was required to identify the emotion from a picture presenting only the eye region. There were 18 trials, in which pictures were presented in a pseudo-random order. This test was scored in terms of the number of correct answers.

For the emotion identification from dynamic faces test (Tardif et al., 2007), students watched videos of a person who tells a story and plays the principal role of this story. Then, they had to identify the emotion that corresponds to the emotion of the character. Students performed 15 trials, and the score for this test was the sum of correct answers.

The faces memory subtest from NEPSY (Korkman, Kirk, and Kemp, 2003) consisted of presenting pictures of faces to students and then asking them to recognize presented faces from a set of pictures. There were two recognition modalities: an immediate recall and a differed recall. To score this test, one point per item correctly recognized was counted, and, then, a global recognition score to assess the immediate and differed abilities of remembering someone’s face was computed.

High-level socio-emotional functioning was assessed with the LEAS-C (Level of Emotional Awareness Scale for Children), the emotional fluency test, the PEPS-C (Profiling Elements of Prosodic Speech for Children in its French version), and the intention attribution test.

The LEAS-C (Bajgar et al., 2005) consisted of presenting short evocative scenarios in mainstream environments (mainly at school) to the student and then asking him to verbally describe his own feelings and the other person’s feelings. Each scenario was presented in two or four sentences and involved two individuals. Scoring was made on the basis of the complexity of the response in terms of the number of adjectives and of the richness of the formulation—there were five levels of awareness from which the test was scored: 1 point for level 1, 2 points for level 2, and so on. Answers for own feelings and for other’s feelings were averaged to obtain a total score.

The emotional fluency test (Greenberg et al., 1995) was used to assess the ability for an individual to verbally express his internal emotional states by measuring his/her access to an emotional lexicon. Students were required to produce a maximal number of words that depicted an emotional state in a restricted time (2 min). This test was scored in terms of the number of correct produced words in the limited time.

The PEPS-C (Peppé et al., 2010) was used to assess the prosodic skills of students. This test was designed to assess grammatical and affective prosody from a perceptive and productive point of view. In this study, the perceptive affect task, which consisted of determining if the speaker likes or does not like a food item based on the intonation of the speaker’s voice, was used. There were 42 trials with three modalities of emotional valence (neutral, positive, and negative). The score was computed from the number of correct answers

The intention attribution task (Brunet et al., 2000) consisted of presenting little comic strips and asking the students to choose the picture that completes the story. Each comic strip was composed of three pictures. This test was scored in terms of number of good answers.

Executive functioning measures: To assess executive attentional functioning, a set of subtests was selected from the French version of the TAP battery (Test for Attentional Performance, Zimmermann and Fimm, 1997), as follows:

Working memory subtest: This was used to assess the control process of entering information flows and their updates in working memory. The student was required to determine for each presented number if this number was the same than the preceding one. To do so, he had to maintain and to update information about the last numbers he stored in working memory. The score for this subtest was determined from the number of identified targets.

Divided attention subtest: This was used to assess the attentional processes that simultaneously distribute attentional resources for two kinds of stimuli (visual and auditory tasks). The student was asked to perform two tasks simultaneously. In the visual task, the student answered when he saw four crosses drawing a square, and in the auditory task, he answered when he heard the same sound two times in a row. This test was scored in terms of averaged reaction time.

Set shifting subtest: This was used to assess the set shifting process, as the cognitive flexibility to move one’s own attention from a stimulus to another depending on environmental demands. The student had two buttons to answer and was required to consequently answer to two kinds of stimuli; he had to first push the button on the side of the letter and then push the button on the side of the number. This test was scored in terms of averaged reaction time.

Go/no-go subtest: This was used to assess inhibitory processes thanks to a task that requires appropriately reacting to a kind of stimuli whilst inhibiting inadequate answers. The student was required to answer to a particular stimulus and not to the others: An addition sign (+) and a multiplication sign (×) were pseudo-randomly displayed and the student had to choose one of them. This test was scored in terms of averaged reaction time.

## Appendix B. Adaptive Behaviors Assessment

The EQCA-VS (Morin and Maurice, 2001) is a francophone parent- and/or teacher-reported assessment of adaptive behaviors at school. This test consists of a questionnaire that assesses both adaptive and problem behaviors of the students on several dimensions. Adaptive behaviors are divided into five dimensions: (1) communication, (2) socialization, (3) autonomy, (4) preschool and school abilities and (5) leisure; maladaptive behaviors refer to violent behaviors, withdrawal behaviors, unacceptable behaviors and habits, antisocial behaviors and inadequate sexual behaviors. Parents and specialized teachers completed the questionnaire by indicating, for each item, if the student exhibits the behavior. We obtained total scores for adaptive and problem behaviors, as well as subscores for each dimension of adaptive behaviors (higher scores revealed a greater occurrence of adaptive or problem behaviors).

The SRS-2 (Constantino and Gruber, 2005) is a parent- and/or teacher-reported 65-items questionnaire designed to quantitatively measure the ability of a child to emotionally engage in reciprocal social interactions This questionnaire flies over social impairments of a child in naturalistic social settings and has often been used in research to estimate the impacts of ASD symptomatology on daily functioning (e.g., Kanne et al., 2011; Kuhlthau et al., 2010; Leung et al., 2016). Indeed, it rates five areas of reciprocal social interactions: (1) social awareness, which assesses the ability to pick up on social cues; (2) social cognition, which assesses the ability to interpret social cues; (3) social communication, which assesses the ability to include expressive social communication; (4) social motivation, which assesses the extent of motivation to engage in social interpersonal behavior; (5) autistic mannerisms, including stereotypies, restricted interests, and repetitive behaviors. Each item is rated from "0" (never true) to "3" (almost always true) to obtain a score between 0 and 195: the higher the score; the more severe the social impairments, and an R-score higher than 60 indicates a high probability of ASD.
